# In-feed organic and inorganic manganese supplementation on broiler performance and physiological responses

**DOI:** 10.5713/ab.20.0797

**Published:** 2021-06-23

**Authors:** Bruno Reis de Carvalho, Hélvio da Cruz Ferreira, Gabriel da Silva Viana, Warley Junior Alves, Jorge Cunha Lima Muniz, Horácio Santiago Rostagno, James Eugene Pettigrew, Melissa Izabel Hannas

**Affiliations:** 1 Department of Animal Science, Universidade Federal de Viçosa, Viçosa, MG 36570900, Brazil; 2 Production Systems, Natural Resources Institute Finland (Luke), Jokioinen 31600, Finland; 3 Pettigrew Research Services, Inc., Champaign, IL 61821, USA

**Keywords:** Mineral Excretion, Organic Trace Minerals, Poultry Nutrition, Tibia Mineralization

## Abstract

**Objective:**

A trial was conducted to investigate the effects of supplemental levels of Mn provided by organic and inorganic trace mineral supplements on growth, tissue mineralization, mineral balance, and antioxidant status of growing broiler chicks.

**Methods:**

A total of 500 male chicks (8-d-old) were used in 10-day feeding trial, with 10 treatments and 10 replicates of 5 chicks per treatment. A 2×5 factorial design was used where supplemental Mn levels (0, 25, 50, 75, and 100 mg Mn/kg diet) were provided as MnSO_4_·H_2_O or MnPro. When Mn was supplied as MnPro, supplements of zinc, copper, iron, and selenium were supplied as organic minerals, whereas in MnSO_4_·H_2_O supplemented diets, inorganic salts were used as sources of other trace minerals. Performance data were fitted to a linear-broken line regression model to estimate the optimal supplemental Mn levels.

**Results:**

Manganese supplementation improved body weight, average daily gain (ADG) and feed conversion ratio (FCR) compared with chicks fed diets not supplemented with Mn. Manganese in liver, breast muscle, and tibia were greatest at 50, 75, and 100 mg supplemental Mn/kg diet, respectively. Higher activities of glutathione peroxidase and superoxide dismutase (total-SOD) were found in both liver and breast muscle of chicks fed diets supplemented with inorganic minerals. In chicks fed MnSO_4_·H_2_O, ADG, FCR, Mn balance, and concentration in liver were optimized at 59.8, 74.3, 20.6, and 43.1 mg supplemental Mn/kg diet, respectively. In MnPro fed chicks, ADG, FCR, Mn balance, and concentration in liver and breast were optimized at 20.6, 38.0, 16.6, 33.5, and 62.3 mg supplemental Mn/kg, respectively.

**Conclusion:**

Lower levels of organic Mn were required by growing chicks for performance optimization compared to inorganic Mn. Based on the FCR, the ideal supplemental levels of organic and inorganic Mn in chick feeds were 38.0 and 74.3 mg Mn/kg diet, respectively.

## INTRODUCTION

Manganese (Mn) is a bioactive element required for multiple postabsorptive physiological processes in poultry, such as carbohydrate, lipid, and amino acid metabolism, as well as cartilage and bone development, and antioxidant defense [1]. For fast-growing broilers, Mn deserves concern since its manipulation in practical feeds may attenuate the incidence of certain disorders associated with fast growth and, in turn, may protect against adverse impacts on revenue and profitability of the chicken meat industry. As a cofactor for glycosyltransferase, Mn acts by attaching glucosamines to a protein core during the synthesis of proteoglycans, an important constituent of bone organic matrix and cartilage [2]. Low concentrations of Mn in broiler feeds have been shown to decrease the activity of glycosyltransferase and, consequently, the synthesis of proteoglycans [3], which is commonly associated with the incidence of leg abnormalities in broilers fed Mn deficient feeds [4,5]. In turn, leg abnormalities in commercial flocks lead to deprivation of locomotive freedom and restricted access to feeds, and consequently to impairments in growth and feed efficiency, which on welfare and economic grounds is undesired in intensive poultry rearing [6].

Manganese is also a component of Mn-containing superoxide dismutase (SOD), an essential antioxidant metalloenzyme responsible for free radical scavenging in mitochondria by catalyzing the dismutation of superoxide anions produced from the electron transport chain to molecular oxygen and hydrogen peroxide [7]. Mitochondria are the major oxidative phosphorylation site where carbohydrates and fats are oxidized to produce energy for cell functioning [8]. Electron and proton leaks across the mitochondrial respiratory chain are believed to increase the generation of free radicals, which may reduce the respiratory chain function or even result in cellular apoptosis [7,8]. Apart from their consequences on the energetic efficiency of cells, intracellular oxidation of lipids and proteins caused by free radicals has a severe practical implication for the poultry meat industry, since damage in tissues affects relevant sensory traits of meat such as overall appearance, color, texture, and flavor [9]. Lu et al [5] demonstrated that dietary Mn supplementation upregulated Mn-SOD gene expression, increased Mn-SOD activity, and reduced malondialdehyde content, a biomarker of lipid peroxidation, in leg muscles of growing chicks.

Manganese requirements of growing broilers are described by the National Research Council [10] based on leg abnormalities and growth as 60 mg/kg diet. This value is half the 120 mg Mn/kg recommended by Lu et al [5] few years earlier. Contrary to NRC [10], the referred authors [11] based their estimates on growth performance, tissue mineralization, and Mn-SOD activity in heart. As an attempt to reevaluate growing chick responses to supplemental Mn, Lu et al [4] found that neither growth performance nor meat quality traits were affected by the supplemental Mn levels under investigation (100 to 500 mg Mn/kg diet as Mn sulphate), indicating that adding 100 mg Mn/kg diet supports broiler carcass traits and meat quality. Even though relevant as references, these findings fail to establish the minimum level of supplemental Mn to optimize chick responses.

From the mid-2000s, organic trace minerals (OTM) received considerable attention due to their potential benefits and advantages compared with inorganic salts. After decades of investments and research efforts, OTM have proved to be more bioavailable than carbonates, oxides, and sulphates traditionally used by industry [12]. One point commonly ignored in assessments of poultry responses to sources and concentrations of trace minerals, which could potentially lead to misinterpretations of outcomes, is the source of the other trace minerals provided in the supplement in experimental diets. Generally, trace mineral supplement used in assays conducted to determine ideal levels of a given OTM provide all the other trace minerals as inorganic salts. Although widespread, such approach might not be the most accurate one to validate optimum levels of organic minerals for poultry. Firstly, because it fails in mimicking the conditions under which commercial flocks are reared. In intensive broiler rearing, Mn provided as inorganic salts has been typically supplemented with other trace minerals also in inorganic form, whereas Mn as proteinates or chelates has been offered in feeds with other OTMs. Therefore, assessment of the optimal dietary concentrations of supplemental manganese for use by the poultry industry may be most appropriately accomplished in the dietary environment in which each manganese source will typically be used in practice, organic manganese with other organic minerals and inorganic manganese with other inorganic minerals.

Secondly, Mn interacts with other trace minerals in many physiological processes either antagonistically or synergistically. A classic antagonistic interaction of Mn has been reported with iron (Fe). Both minerals share two common transport protein in intestine, the cellular importer divalent metal transporter 1 (DMT1) and the cellular exporter ferroportin 1 [13,14]. Evidences have suggested that higher amounts of Fe inhibit the expression of DMT1 in enterocytes, and lead to a depression of Mn uptake [15]. Because OTMs may be absorbed as peptides and/or amino acids in intestine, it seems reasonable to hypothesize that organic Fe in feeds, for example, could optimize the absorption and utilization of organic Mn by broilers. Therefore, chick responses to organic Mn could be potentially different, and lower levels could be required whether all the trace mineral sources are provided in organic form rather than inorganic form. We hypothesized that the supplemental level of organic Mn required to optimize growing broiler chick responses would be lower than inorganic supplemental Mn. Therefore, we conducted an experiment to evaluate the effects of supplemental levels of Mn provided by organic and inorganic trace mineral (ITM) supplements on growth, tissue mineralization, mineral balance, and antioxidant status of growing broiler chicks.

## MATERIALS AND METHODS

### Animal care

Animals care and use procedures described in this section were in accordance with Brazilian Legislation on Animal Experimentation and Welfare, and the experimental protocol was approved by the Ethics Committee on the Use of Farm Animals (CEUAP-UFV) of the Universidade Federal de Viçosa, (protocol 111/2014) before the initiation of the trial.

### Birds and husbandry

A total of five hundred 1-d-old male Cobb 500 chickens obtained from a local commercial hatchery were used in this study. During the first seven days of age, the birds were fed a pre-starter diet based on corn and soybean meal formulated to meet or exceed nutritional recommendations of Rostagno et al [16], except for Mn, whose dietary concentration was provided at 43 mg Mn/kg of feed as manganese sulphate (MnSO_4_·H_2_O), which corresponded to 50% of the dose recommended by NRC [10]. The pre-starter diet contained less Mn than typically used in commercial production to avoid excessive storage reserves of this mineral. Throughout the entire pre-experimental period, chicks had free access to water and mash feed. On d 8 post hatch, chicks were housed in an environmentally controlled room and allotted into 49 cm× 27 cm×33 cm (length×height×width) plastic cages with raised wire floors until the end of the feeding assay. Chicks were allowed *ad libitum* access to feed and demineralized water throughout the 10-d experimental period via plastic feeders and cup drinkers. Photoperiod was set at 12 h natural light/12 h artificial light. Both temperature and humidity were set according to genetic guideline recommendations.

### Experimental diets and treatments

Prior to the experimental period, all chicks were weighed and assigned to treatment groups so that initial body weight (BW, 174.6±1.0 g) was similar among experimental treatments. A 2×5 fractional factorial arrangement design was used in this study, where two Mn sources were added to basal Mn deficient diets at the supplemental Mn levels of 0, 25, 50, 75, and 100 mg Mn/kg diet. The organic source of Mn under study was manganese proteinate (MnPro, 13.9% Mn, Bioplex Mn, Alltech, São Pedro do Ivaí, Brazil), whereas inorganic Mn was supplied as manganese sulphate (MnSO_4_·H_2_O, 30.3% Mn). Chicks were randomly assigned to ten treatments with 10 replicates of five birds per treatment. A semi purified basal diet was formulated to meet or exceed Rostagno et al [16] recommendations for starting broilers (8 to 21 days of age), except for trace minerals. The basal diet was based on casein, albumin, isolated soybean protein, corn, starch, and dextrose (Table 1), from which 4 different diets were produced. The 4 diets differed from each other with regard to amount and source of Mn supplemented (MnPro or MnSO_4_·H_2_O) and the source of other trace minerals (organic or inorganic) as follows: i) basal diet supplemented with OTM supplement without supplemental Mn; ii) OTM+100 mg Mn/kg feed supplemented as MnPro; iii) basal diet supplemented with ITM supplement without supplemental Mn; and iv) ITM + 100 mg Mn/kg of feed supplemented as MnSO_4_·H_2_O. Diets supplemented with OTM containing 0 and 100 mg Mn/kg as MnPro were mixed in appropriate ratios to produce five diets with supplemental organic Mn concentrations of 0, 25, 50, 75, and 100 mg Mn/kg of feed. The same procedure was adopted with diets supplemented with ITM, to obtain the five levels of manganese. The OTMs were supplied as Bioplex Fe (15% Fe), Bioplex Zn (15.0% Zn), Bioplex Cu (11.4% Cu), and selenium yeast as Selplex (0.236% Se), whereas ITMs sources included iron sulphate (21.9% Fe), zinc sulphate (22.9% Zn), copper sulphate (25.1% Cu), and sodium selenite (51.6% Se) in ITM, and iodine was added as calcium iodide (86% I) in both trace mineral supplements. Except for Mn, all trace minerals were supplied to meet or exceed NRC [10] recommendations. Sodium phytate and cellulose were added to the semi-purified diet to simulate practical cereal-based-diets. All test diets were supplemented with a commercial microbial phytase enzyme. Prior to the beginning of the assay, diets were analyzed for Mn concentration (Table 2) as described by AOAC [17]. Overall, the analyzed dietary concentrations of Mn were close to those calculated.

### Performance, sampling, and chemical analysis

On d 17 post-hatch, all chicks and feed leftovers from each experimental unit were weighed to determine the final BW, ADFI, average daily gain (ADG), and feed conversion ratio (FCR). From d 13 to 17 post-hatch, trays covered with plastic were placed underneath pens for total excreta collection. Total excreta were collected daily, weighed, and frozen (−20°C). At the end of the collection period, excreta from each pen were homogenized. On d 18 post-hatch, one bird per cage (10 birds/treatment) was randomly selected and sacrificed by cervical dislocation. Subsequently, the breast, liver, and left and right tibia were collected and stored at −20°C. The bird had the breast muscle, liver, and left and right tibia collected, stored at −20°C, and subsequently, were ether extracted for 4 h in a Soxhlet extractor. Posteriorly, these samples, as well the samples of excreta, were freeze-dried for 72 h at −80°C under 800 mbar of pressure (Liobras, São Carlos, SP, Brazil), ground in a stainless ball mill (Micro spray mill, R-TE 350, TECNAL, Ourinhos, SP, Brazil), and finally analyzed in the atomic absorption spectrophotometer (Spctr AA-800; Varian spectrometer, Harbor City, CA, USA) at Animal Nutrition Laboratory (Universidade Federal de Viçosa, Viçosa, MG, Brazil) to obtain the Mn, Cu, Zn, Fe, Ca, and P concentrations as described by AOAC [17]. Manganese retention was calculated through the difference between the amount of Mn consumed and excreted and expressed as mg/kg of BW. The total superoxide dismutase (total-SOD) and glutathione peroxidase (GSH-Px) activity in breast muscle, and liver tissues were performed according to Walsh et al [18] using the kits of Randox Laboratories Ltd. (County Antrim, UK) Ransod and Ransel, respectively, through an automatic biochemical analyzer (Mindray BS-200E, Shenzhen Mindray Bio-Medical Electronics Co., Shenzhen, China) following the manufacturer guidelines.

### Statistical analysis

Data were analyzed as a completely randomized design under an incomplete 2-way (source×levels) factorial assay with inorganic and OTMs supplement without Mn supplementation (zero level) and Mn supplement levels as organic Mn in OTM supplement and inorganic Mn in ITM supplement. In this context, the traditional factorial analysis was updated to an incomplete factorial design [19]. This approach is easily accomplished by using common statements from PROC MIXED of SAS (Version 9.4, SAS Institute Inc., Cary, NC, USA) software. According to the previously mentioned analysis, the significance (p<0.05) of source effect (only two levels) was evaluated through F-test; whereas orthogonal contrasts were applied to perform the analysis between linear and quadratic responses of dependent variables in function of increasing Mn levels. Additionally, when Mn levels in each source were significant, means were compared using Tukey’s multiple comparison test. Quadratic polynomial regression model is:

$$\text{Y}=\beta 0\times \beta 1\times \text{X}+\beta_{2}\times \text{X}2,$$

where Y is the dependent variable, X is the dietary Mn concentration, and β0 is the intercept, β1 and β2 are the linear and quadratic coefficients, respectively. Additionally, performance data were fitted to linear broken-line model (LBL) using NLMIXED procedure of SAS (Version 9.4, SAS Institute Inc., Cary, NC, USA) software to estimate the supplemental Mn level which optimized performance responses assessed. The LBL model was expressed as:

$$\text{Y}=\beta 0+\beta 1\times (\beta_{2}-\text{X});\text{where }(\beta 2-\text{X})=0\;\text{for X}>\beta 2,$$

where Y is the dependent variable, X is the dietary Mn concentration, β0 is the value at the plateau, β1 is the slope and β2 is the Mn supplemental at the break point (level to optimize the biological response). Statistical significance was considered as 0.05 for all executed analysis. The term “tendency” was used for situations in which p-value is between 0.05 and 0.10.

## RESULTS

For both Mn sources under study, the analyzed values of Mn in experimental diets were close to those expected, as well as the concentrations of Fe, Cu, and Zn (Table 2).

### Performance responses

Neither interactive effects between sources and supplemental Mn levels (p>0.05) nor main effects of sources (p>0.05) were noticed on chick performance responses assessed (Table 3). Chicks fed diets supplemented with Mn (25 to 100 mg/kg diet) exhibited higher BW, ADG, and better FCR (p<0.05) compared with chicks fed the basal diet with no supplemental Mn. A tendency for higher (p = 0.078) ADFI was noticed on the group of chicks fed Mn supplemented diets. Manganese supplementation improved (p<0.05) linearly all the performance traits assessed and a tendency of quadratic effect of the levels under study was noticed for BW (p = 0.072) and ADG (p = 0.070). The BW and ADG increased (p<0.05) linearly as dietary Mn supplementation as MnPro or MnSO_4_·H_2_O (Table 4) increased. Chick FCR decreased (p<0.05) linearly as dietary MnSO_4_·H_2_O supplementation increased and decreased (p<0.05) linearly and were quadratically (p< 0.05) affected by increasing dietary MnPro supplementation (Table 4).

### Manganese balance

Manganese supplemental levels and sources influenced the intake, excretion, retention, and balance of Mn in broiler chicks (Table 5). No interaction effects (p>0.05) between sources and levels of Mn were noticed on Mn balance responses. Chicks fed MnPro supplemented diets exhibited a lower intake of Mn (p<0.05) compared with MnSO_4_·H_2_O fed chicks. Chick intake, excretion, and retention of Mn increased (p<0.05) gradually in each supplemental Mn level, achieving the highest values at 100 mg Mn/kg diet. Manganese intake, excretion, and retention were linearly increased (p<0.05) as supplementation Mn levels increased. Chicks fed diets without supplemental Mn exhibited the lowest Mn balance (p<0.05) compared with the other Mn supplemental levels. Manganese balance was linearly and quadratically influenced (p<0.05) by Mn supplementation. Chicks fed dietary MnPro or MnSO_4_·H_2_O supplementation levels exhibit a linear increase (p<0.05) in Mn intake, Mn excretion, and Mn retention (p<0.05) (Table 4). Chicks fed basal diets without supplemental Mn showed a Mn balance strikingly lower (p< 0.05) compared with MnPro or MnSO_4_·H_2_O fed chicks. Both linear and quadratic effects of MnPro and MnSO_4_·H_2_O supplementation were noticed on chick Mn balance (p<0.05).

### Manganese tissue concentrations

No interactive effects between Mn source and Mn supplementation levels (p>0.05) were noticed on Mn tissue concentration (Table 6). The highest concentrations of Mn (p<0.05) in liver, breast muscle, and tibia were noticed at 50, 75, and 100 mg Mn/kg diet, respectively. Chicks fed basal, i.e. without supplemental Mn, exhibited the lowest Mn concentrations (p< 0.05) in the tissues. Manganese supplementation elicited a linear increase (p<0.05) in Mn tissue concentrations in breast muscle, liver, and tibia. A quadratic response to dietary supplemental Mn levels (p<0.05) was noticed for Mn concentration in liver and tibia of chicks, whereas a tendency of quadratic effect (p = 0.074) of levels was noticed for Mn content in breast muscle. Manganese concentration in breast, liver, and tibia were increased (p<0.05) linearly and quadratically in response to increased dietary MnPro supplementation (Table 4). Dietary MnSO_4_·H_2_O supplementation elicited a linear increase (p<0.05) in Mn concentration in liver and tibia and quadratic increase (p<0.05) in liver (Table 4).

### Antioxidant enzyme activity

As detailed in Table 7, no interactive effects between source and supplemental Mn levels were noticed (p>0.05) on antioxidant enzyme activity in chick liver and breast muscle (Table 7). Chicks fed MnSO_4_·H_2_O supplemented diets exhibited a higher activity of GSH-Px in the liver and breast muscle (p< 0.05) compared with MnPro fed chicks. Breast muscle total-SOD activity tended to be higher (p = 0.081) in MnSO_4_·H_2_O fed chicks. The activity of GSH-Px in breast muscle tended (p = 0.074) to increase linearly as dietary MnPro supplementation increased (Table 4). GSH-Px activity in liver tended to be quadratically affected (p = 0.089) by MnSO_4_·H_2_O supplementation.

### Optimal supplemental Mn levels for organic and inorganic sources

Data of performance, Mn balance, and Mn tissue concentration were fitted to different regression models as summarized in Table 8. The supplemental Mn levels which optimized performance traits assessed according to LBL regression model are detailed in Table 8. When fitting data collected from both sources to the LBL model, the optimum levels of Mn for BW and ADG was 27.6 mg Mn/kg diet, whereas FCR was optimized at 61.3 mg Mn/kg diet. As MnPro, Mn supplemental levels for optimum BW, ADG, and FCR were estimated as 21.6, 20.6, 38.0 mg Mn/kg diet, respectively. Chicks fed MnSO_4_·H_2_O supplemented diets had the BW, ADG, and FCR optimized at supplemental Mn level of 59.9, 59.8, and 74.3 mg Mn/kg diet, respectively. Manganese balance achieved its maximum value at the supplemental level of 18.6 mg Mn/kg diet. When different Mn sources were considered in regression analysis, chicks fed MnSO_4_·H_2_O and MnPro supplemented diets had Mn balance maximized at 20.6 and 16.6 mg/kg diet, respectively. The greatest concentration on Mn in breast muscle and liver were observed at the supplemental level of 67.5 and 39.3 mg Mn/kg diet, respectively. Chicks fed MnPro supplemented diets exhibited maximum Mn concentration at breast and liver at the supplemental Mn level of 62.3 and 33.5 mg/kg, respectively. For chicks fed MnSO_4_·H_2_O diets maximum Mn concentration in liver were achieved at the supplemental level of 43.1 mg Mn/kg.

## DISCUSSION

Establishing broiler requirements for Mn has been revealed to be particularly challenging, since estimates and biological responses may be affected by several factors, which include the source of Mn under study, the experimental basal diets used to produce treatments, the concentration, and source of the other trace minerals in experimental diets, as well as the biological response used as a reference to determine the optimal level. In the current research, a semi-purified diet based on dextrose, casein, and albumin was supplemented with Mn levels and sources to produce dietary treatments. As highlighted in the last revised edition of Nutrient Requirements of Poultry [10], estimates of trace mineral requirements of chicks fed semi-purified diets are expected to be lower than those obtained from cereal-based diets due to poor or nonexistent presence of antinutritional factors such as phytate and fiber. In this experiment, both sodium phytate and cellulose, as well as a microbial phytase, were added to experimental diets to simulate a commercial cereal-based diet. When investigating growing chick requirements for Mn using a semi-purified dextrose-casein diet, Southern and Baker [20] estimated optimal Mn level at 14 mg Mn/kg diet. In the current research, with the same type of ingredients, we estimated higher requirements, whose values were similar to literature. Such fact indicates fiber and sodium phytate added to experimental diets fulfilled the purpose of simulating practical cereal-based diets.

Our outcomes demonstrated that regardless of the sources assessed, performance traits were influenced by Mn supplementation to the basal feeds, which proves the essentiality of this mineral for growing chicks. Even though chicks fed 25 mg supplemental Mn/kg diet achieved similar performance targets to chicks fed the highest Mn level under study, there was a linear improvement in performance responses as Mn supplementation increased, mainly in FCR (Table 3). In order to describe responses to supplemental Mn; and estimate the supplemental Mn levels required to optimize the performance traits, ADG, and FCR data were fitted to the LBL regression model. As summarized in Table 8, in chicks fed MnSO_4_·H_2_O supplemented diets, ADG increased linearly and reached the plateau at 59.8 mg Mn/kg diet, whereas FCR decreased linearly and achieved a flat line at 74.3 mg Mn/kg. In turn, MnPro fed chicks had the ADG and FCR optimized at 20.6 and 38 mg Mn/kg diet. Our estimates for the inorganic Mn level suggest that NRC [10] recommendations may support ADG, but higher concentrations are required for FCR optimization.

As expected, our findings clearly demonstrate that com pared with MnSO_4_·H_2_O, lower amounts of Mn were required to reach maximal chick performance when Mn was provided as MnPro via the OTM supplement. So far as the mechanisms underlying Mn absorption are understood, Mn uptake occurs mainly in the upper small intestine by the transport protein DMT1 [21,22], whose expression in intestinal mucosa is modulated by Mn source and dietary level [13]. Previous findings have demonstrated that complexed or chelated organic Mn increases mRNA expression of DMT 1 in broiler chick small intestine compared with Mn sulphate [23], which explains, at least in part the fact that lower levels of organic Mn were able to reproduce similar performance to higher levels of inorganic Mn in the current research.

Previous reports have suggested that Mn supplementation did not affect ADG and FCR of growing chicks, and that cereal-based diets containing 19 to 26 mg Mn/kg from cereal grains in the diet without a supplemental Mn source, could support performance objectives [1,3,4]. Our findings indicate that 25 mg supplemental Mn/kg diet was sufficient to support proper growth rates, which considering Mn content in basal diet, i.e. 6 mg Mn/kg, would be equivalent to 31 mg Mn/kg. Despite similarities, a comparison between our requirement estimates and those described in the refereed references cannot be made with confidence due to differences in the Mn sources. The bioavailability of Mn sources is limited in poultry, especially in cereals [10]. Wedekind et al [24] reported that only 9% of the Mn provided by a corn and soybean-based diet supplemented with MnSO_4_·H_2_O (100 mg Mn/kg diet) was absorbed by broiler chicks. Yet, the authors reported that such rate was 2.8% when no Mn source was added to basal diet. Such findings may be clearly supported by our results. As detailed in Table 5, the amount of Mn retained in chicks relative to Mn intake, i.e. Mn balance, was 56% lower in birds fed basal diets compared with the balance in chicks fed the lowest supplemental Mn level of 25 mg/kg diet. Even though Mn balance differed among Mn-supplemented groups, the difference was narrow between the lowest and highest Mn balance (35.5% vs 36.5%), which suggests that dietary levels higher than those estimated for performance optimization were utilized by chicks and retained in the body, as our data for tissue mineralization show. As detailed in Table 8, when fitting Mn balance data from chicks fed both Mn sources to LBL regression model, slightly lower levels were estimated maximum Mn balance (18.6 mg Mn/kg). As expected, a lower supplemental Mn level was estimated to maximize Mn balance in chicks fed MnPro (16.6 Mn/kg) compared with MnSO_4_·H_2_O fed chicks (20.6 mg Mn/kg).

Manganese levels influenced liver Mn concentrations; whose greatest value was at 50 mg supplemental Mn/kg diet. Despite being statistically different, the means of liver Mn content from chicks fed 50 and 100 mg Mn/kg diet were quite similar numerically (11.5 vs 11.3 mg Mn/kg). Likewise, after adding tribasic manganese chloride or MnSO_4_·H_2_O to a low Mn basal diet, Conly et al [25] reported that chick liver Mn content increased up to 60 mg Mn/kg diet and remained constant up to 130 mg Mn/kg diet, regardless of the source assessed. When fitting liver Mn concentration data to LBL model, the breakpoints for maximum concentration were estimated at 39.3 mg Mn/kg diet considering both sources, and 33.5 and 43.1 mg Mn/kg diet for chicks fed MnPro and MnSO_4_·H_2_O supplemented diets, respectively. Liver is the primary organ responsible for regulating Mn body status through biliary excretion [26]. When provided in concentrations that exceed physiological needs, Mn may be progressively accumulated in different organs and, beyond critical limits, be excreted to avoid toxicity [20]. Chicks fed higher levels of Mn than the requirements established herein for organic (38 mg Mn/kg diet) and inorganic (74.3 mg Mn/kg diet) stored Mn in extra-hepatic tissues (e.g. breast and tibia). Breast muscle Mn content responded with increasing Mn deposition up to 75 mg Mn/kg diet, whereas Mn deposition in tibia continuously increased up to 100 mg Mn/kg diet, the highest level under study. Our findings support those reported by Yan and Waldroup [27] who reported that regardless of the source of Mn assessed (MnO, MnSO_4_·H_2_O, or amino acid chelated Mn), Mn concentrations in broiler tibia were gradually increased up to 800 mg supplemental Mn/kg diet. Similarly, when investigating the Mn supplementation on broiler diets, Conly et al [25] noticed that Mn was continuously deposited in the tibia of chicks fed tribasic manganese chloride or MnSO_4_·H_2_O supplemented diets (0, 30, 60, and 130 mg supplemental Mn/kg), achieving the greatest value at the highest level studied. Although Mn content in breast muscle and tibia were unaffected by Mn sources, the analysis of levels in each source suggests that Mn concentration in breast increased until 75 mg Mn/kg only when Mn was supplemented as MnPro. According to polynomial regression model estimates, Mn concentration in breast reached its maximum values at supplemental Mn level of 67.5 mg/kg, and for chicks fed MnPro, maximum deposition was achieved at 62.3 mg/kg. We noticed that Mn concentrations in tibia and liver approximately doubled in chicks fed the highest supplemental Mn level compared with chicks fed diets without supplemental Mn. Such outcomes differ from those reported by Lu et al [5] that neither amino acid chelated Mn nor MnSO_4_·H_2_O affected breast muscle Mn content of growing chicks fed diets supplemented at 100 and 200 mg Mn/kg, and from those reported by Yan and Waldroup [27] who observed a higher Mn content in the tibia of chicks fed amino acid chelated Mn compared with chicks fed MnO and MnSO_4_·H_2_O. Our results suggest that Mn concentration in breast muscle are mainly influenced by proteinate Mn source, i.e. MnPro.

Although Mn levels did not affect antioxidant enzyme activity, we observed that, curiously, chicks fed inorganic minerals supplemented diets exhibited higher activity of GSH-Px in breast muscle and liver, and higher total-SOD activity in liver compared with chicks fed organic minerals. Organic minerals are potentially more bioavailable than the inorganic forms, so it was expected that they would support higher total-SOD activity. It is worth highlighting, however, that although trace minerals modulate the activity of antioxidant enzymes like SOD, GSH-Px, and catalase, they may also act as pro-oxidant agents [28,29]. Free iron and copper, for example, have been described as the major catalyzers of the production of free radicals such as hydrogen peroxide (H_2_O_2_) and hydroxyl, which disrupt the redox balance in cells, and cause oxidative damage to tissues [30,31]. Because ITMs are affected by the variation of pH, they may reach some tissues such as gut mucosa and blood as reactive ions, which may potentially oxidize cytosolic structures and DNA of cells. Therefore, inorganic minerals may be potentially pro-oxidant compared with OTMs, whose chemical structures are more stable and not so easily dissociated. Even though GSH-Px is an enzyme dependent on selenium and not Mn, its activity is expected to increase in response to the increase in total-SOD activity. Whereas SOD acts in a first level, in the dismutation of superoxide radical to H_2_O_2_, GSH-Px ends the process by detoxifying H_2_O_2_.

## CONCLUSION

To the best of our knowledge, this is the first research where the ideal level of organic Mn for broiler chicks was estimated using a supplement in which the other trace minerals (zinc, copper, iron, and selenium) were provided as organically complexed metals. As hypothesized, when compared with inorganic Mn, lower levels of organic Mn are required by growing chicks for performance optimization. Overall, estimates of supplemental Mn levels required to optimize Mn balance and Mn tissue concentration in chicks were lower than those levels to optimize feed conversion ratio in chicks. Based on the feed conversion response, the ideal supplemental levels of organic and inorganic Mn for broiler chicks were 38.0 and 74.3 mg Mn/kg diet, respectively.

## Figures and Tables

**Table 1:** Ingredient and composition of the semi-purified basal diet (as-fed basis)

Items	
Ingredients (g/kg)	
Corn	300
Dextrose	133
Starch	130
Albumin^1)^	120
Broken rice	80
Cellulose^1)^	40
Casein^1)^	40
Soybean protein isolate	40
Soybean oil	20
Calcium carbonate^1)^	16.95
Potassium phosphate^1)^	14.85
Magnesium chloride^1)^	6.50
Potassium chloride^1)^	4.68
Choline chloride, 60%	3.75
Mixture of amino acids^2)^	35.55
Micronutrients^3)^	12.20
Microminerals^4)^	2.00
Phytase^5)^	0.10
Calculated nutrients	
AMEn (kcal/kg)	3,128
Crude protein^6)^ (%)	22.58
SID amino acids (%)	
Lysine (%)	1.25
Methionine (%)	0.55
Methionine + cystine (%)	0.91
Threonine (%)	0.83
Calcium^6)^ (%)	0.85
Total P^6)^ (%)	0.56
Available P (%)	0.40
Mn^6)^ (mg/kg)	6.0

1) P.A purism reagent exceeds standard ACS specification in trace metals analysis.

2) 0.03% L-lysine (79%); 0.27% L-arginine (98.5%); 0.40% L-glycine (98.5%); 0.85% L-alanine (99%); and 2.0% L-glutamic acid (100%). The amino acids alanine, glycine, and glutamic acid were added to maintain the ratio of essential nitrogen to total nitrogen at 0.50.

3) 0.055% coccidiostat; 0.010% avilamycin; 0.030% BHT; 1.02% sodium phytate; and 0.150% vitamin blend supplemented per kg of feed: vitamin A, 9,750 IU; vitamin D_3_, 2,470 IU; vitamin E, 36.6 IU; vitamin B_1_, 2.6 mg; vitamin B_2_, 6.5 mg; vitamin B_6_, 3.64 mg; vitamin B_12_, 15 mcg; vitamin K, 1.95 mg; nicotinic acid, 39.0 mg; pantothenic acid, 13.0 g; folic acid, 0.91 mg; and biotin, 0.091 mg.

4) Mineral blend supplemented per kg of feed: 80 mg Fe; 10 mg Cu; 40 mg Zn; 0.3 mg Se; and 1 mg I. Except for Mn added according each experimental treatment as 0; 25; 50; 75; and 100 mg Mn. Inorganic micromineral sources: ferrous sulphate (21.9% Fe); zinc sulphate (22.9% Zn); copper sulphate (25.1% Cu); sodium selenite (51.6% Se); calcium iodide (86% I); and manganese sulphate (30.3% Mn); and organic micromineral sources: Bioplex®Fe (15% Fe); Bioplex®Zn (15.05% Zn); Bioplex®Cu (11.4% Cu); selenium yeast (0.236% Se); calcium iodide (86% I); and Bioplex® Mn (13.9% Mn).

5) Microbial phytase, 600 FTU/kg.

6) Value determined by analysis. Each value based on 10 replicates.

**Table 2:** Analyzed concentration of manganese and trace minerals in experimental diets (as- fed basis)

Trace mineral sources	Trace minerals^1)^	Manganese supplementation in basal diet^2)^ (mg/kg)				

		0	25	50	75	100
Organic^3)^	Mn	6	27	49	70	93
	Fe	108	104	105	107	111
	Cu	12	13	12	13	12
	Zn	51	51	56	53	52
Inorganic^3)^	Mn	6	27	53	73	96
	Fe	113	103	109	113	97
	Cu	6	5	6	6	8
	Zn	67	73	66	73	69

1) Value determined by analysis. Each value based on 10 replicates.

2) Manganese supplementation levels were obtained by adding 0, 25, 50, 75, and 100 mg Mn/kg diet to a semi-purified diet containing 6.0 mg Mn/kg.

3) Organic, manganese proteinate (MnPro) and organic trace minerals; Inorganic, manganese sulphate (MnSO_4_·H_2_O) and inorganic trace minerals.

**Table 3:** Growth performance of broiler chicks fed different supplemental manganese levels of provided by organic and inorganic sources

Items	Manganese levels (mg/kg)					Source^1)^		SEM	p-value				
		
	0	25	50	75	100	Inorganic	Organic		Source	Level	Source ×level	L	Q
BW (g/bird)	526.6^b^	547.7^a^	548.2^a^	547.5^a^	554.2^a^	544.4	545.3	2.15	0.821	<0.01	0.361	<0.01	0.072
ADG (g/bird/d)	35.17^b^	37.29^a^	37.33^a^	37.26^a^	37.93^a^	36.95	37.04	0.214	0.821	<0.01	0.355	<0.01	0.069
ADFI (g/bird/d)	49.86	51.59	51.05	51.24	51.94	51.29	50.98	0.244	0.518	0. 078	0.518	0.027	0.507
FCR (g/g)	1.419^a^	1.390^b^	1.382^b^	1.375^b^	1.365^b^	1.388	1.385	0.039	0.690	<0.01	0.565	<0.01	0.152

SEM, standard error of means; L, linear effect of dietary manganese supplementation; Q, quadratic effect of dietary manganese supplementation; BW, body weight; ADG, average daily gain; ADFI, average daily feed intake; FCR, feed conversion ratio.

1) Organic, manganese proteinate (MnPro) and organic trace minerals; inorganic, manganese sulphate (MnSO_4_·H_2_O) and inorganic trace minerals.

a,b Means with a different superscript in a row are different (p<0.05).

**Table 4:** Linear and quadratic effects of manganese levels supplemented from different sources

Items	Source^1)^	Manganese levels (mg/kg)					p-value^2)^		
	
		0	25	50	75	100	Level	L	Q
BW (g/bird)	MnSO_4_·H_2_O	526.1^b^	542.1^ab^	547.2^ab^	554.5^a^	552.0^a^	0.015	0.002	0.138
	MnPro	527.0^b^	553.3^a^	549.1^ab^	540.4^ab^	556.4^a^	0.009	0.022	0.284
ADG (g/bird/d)	MnSO_4_·H_2_O	35.12^b^	36.73^ab^	37.24^ab^	37.96^a^	37.71^a^	0.014	0.001	0.133
	MnPro	35.22^b^	37.84^a^	37.43^ab^	36.56^ab^	38.16^a^	0.008	0.021	0.282
ADFI (g/bird/d)	MnSO_4_·H_2_O	49.89	51.04	51.63	51.88	52.01	0.271	0.037	0.399
	MnPro	49.83	52.14	50.47	50.59	51.87	0.161	0.294	0.926
FCR	MnSO_4_·H_2_O	1.421^a^	1.391^ab^	1.389^ab^	1.366^b^	1.381^b^	0.003	0.001	0.166
	MnPro	1.417^a^	1.389^ab^	1.374^b^	1.384^ab^	1.360^b^	0.003	0.007	0.047
Intake Mn (mg/kg BW)	MnSO_4_·H_2_O	0.473^e^	2.87^d^	5.19^c^	7.60^b^	10.1^a^	<0.001	<0.001	0.278
	MnPro	0.474^e^	2.84^d^	5.18^c^	7.55^b^	9.99^a^	<0.001	<0.001	0.441
Excreted Mn (mg/kg BW)	MnSO_4_·H_2_O	0.411^e^	1.82^d^	3.26^c^	4.98^b^	6.47^a^	<0.001	<0.001	0.308
	MnPro	0.388^e^	1.87^d^	3.35^c^	4.66^b^	6.42^a^	<0.001	<0.001	0.354
Retention Mn (mg/kg BW)	MnSO_4_·H_2_O	0.062^e^	1.05^d^	1.93^c^	2.62^b^	3.60^a^	<0.001	<0.001	0.593
	MnPro	0.085^e^	0.976^d^	1.76^c^	2.90^b^	3.50^a^	<0.001	<0.001	0.568
Mn balance (%)	MnSO_4_·H_2_O	13.1^e^	36.7^b^	37.1^a^	34.5^d^	35.7^c^	<0.001	<0.001	<0.001
	MnPro	18.0^e^	34.3^d^	34.4^c^	38.4^a^	35.8^b^	<0.001	<0.001	<0.001
Breast muscle Mn (mg/kg DM)	MnSO_4_·H_2_O	0.542	0.575	0.602	0.602	0.597	0.925	0.426	0.610
	MnPro	0.512^e^	0.541^d^	0.702^b^	0.726^a^	0.619^c^	0.016	0.021	0.044
Liver Mn (mg/kg DM)	MnSO_4_·H_2_O	5.59^e^	8.66^d^	12.1^a^	11.4^b^	10.5^c^	<0.001	<0.001	<0.001
	MnPro	6.26^e^	10.0^d^	10.9^c^	11.0^d^	12.0^a^	<0.001	<0.001	0.011
Tibia Mn (mg/kg DM)	MnSO_4_·H_2_O	3.62^e^	4.49^d^	5.50^c^	5.76^b^	6.72^a^	<0.001	<0.001	0.526
	MnPro	3.41^e^	5.20^d^	5.43^c^	5.67^b^	6.44^a^	<0.001	<0.001	0.025
Breast muscle total-SOD (g/g pro)	MnSO_4_·H_2_O	2.10	2.08	2.25	2.19	2.12	0.703	0.648	0.295
	MnPro	2.11	2.05	2.02	2.09	2.00	0.889	0.502	0.948
Liver total-SOD (g/g pro)	MnSO_4_·H_2_O	394	393	401	361	402	0.867	0.870	0.760
	MnPro	412	391	380	399	393	0.962	0.749	0.598
Breast muscle GSH-Px (IU/g pro)	MnSO_4_·H_2_O	1,499	1,493	1,523	1,536	1,521	0.988	0.662	0.876
	MnPro	1,350	1,373	1,356	1,490	1,471	0.341	0.074	0.772
Liver GSH-Px (IU/g pro)	MnSO_4_·H_2_O	31,265	30,428	30,764	30,489	31,539	0.420	0.696	0.089
	MnPro	29,766	30,641	29,924	30,626	29,791	0.515	0.982	0.280

L, linear effect of dietary manganese supplementation; Q, quadratic effect of dietary manganese supplementation; BW, body weight; ADG, average daily gain; ADFI, average daily feed intake; FCR, feed conversion ratio; DM, dry matter; total-SOD, total superoxide dismutase; GSH-Px, glutathione peroxidase.

1) Organic, manganese proteinate (MnPro) and organic trace minerals; inorganic, manganese sulphate (MnSO_4_·H_2_O) and inorganic trace minerals.

2) Significant effect is considered as p<0.05, and 0.05<p<0.10 as tendency.

a–e Means with a different superscript in a row are different (p<0.05).

**Table 5:** Manganese balance in broiler chicks fed different supplemental manganese levels of provided by organic and inorganic sources

Item	Manganese levels (mg/kg)					Source^1)^		SEM	p-value				
		
	0	25	50	75	100	Inorganic	Organic		Source	Level	Source ×level	L	Q
Intake (mg/kg BW)	0.473^e^	2.86^d^	5.18^c^	7.58^b^	10.0^a^	5.24	5.21	0.342	<0.01	<0.01	0.956	<0.01	0.193
Excreted (mg/kg BW)	0.400^e^	1.84^d^	3.31^c^	4.82^b^	6.45^a^	3.39	3.34	0.217	0.108	<0.01	0.405	<0.01	0.170
Retention (mg/kg BW)	0.074^e^	1.015^d^	1.845^c^	2.76^b^	3.55^a^	1.853	1.844	0.128	0.897	<0.01	0.271	<0.01	0.434
Balance (%)	15.6^b^	35.5^a^	35.8^a^	36.5^a^	35.8^a^	31.4	32.2	1.056	0.585	<0.01	0.269	<0.01	<0.01

SEM, standard error of means; L, linear effect of dietary manganese supplementation; Q, quadratic effect of dietary manganese supplementation; BW, body weight.

1) Organic, manganese proteinate (MnPro) and organic trace minerals; inorganic, manganese sulphate (MnSO_4_·H_2_O) and inorganic trace minerals.

a–e Means with a different superscript in a row are different (p<0.05).

**Table 6:** Manganese concentration (dry matter) on tissues of broiler chicks fed different supplemental manganese levels of provided by organic and inorganic sources

Item	Manganese levels (mg/kg)					Source^1)^		SEM	p-value				
		
	0	25	50	75	100	Inorganic	Organic		Source	Level	Source ×level	L	Q
Breast muscle (mg/kg)	0.527^e^	0.558^d^	0.652^b^	0.664^a^	0.608^c^	0.583	0.620	0.017	0.291	0.047	0.445	0.029	0.074
Liver (mg/kg)	5.93^e^	9.35^d^	11.5^a^	11.2^c^	11.3^b^	9.60	10.0	0.295	0.270	<0.01	0.215	<0.01	<0.01
Tibia (mg/kg)	3.52^e^	4.85^d^	5.47^c^	5.72^b^	6.59^a^	5.22	5.23	0.127	0.932	<0.01	0.235	<0.01	0.041

SEM, standard error of means; L, linear effect of dietary manganese supplementation; Q, quadratic effect of dietary manganese supplementation.

1) Organic, manganese proteinate (MnPro) and organic trace minerals; inorganic: manganese sulphate (MnSO_4_·H_2_O) and inorganic trace minerals.

a–e Means with a different superscript in a row are different (p<0.05).

**Table 7:** Antioxidant enzymes activity on breast muscle and liver of broiler chicks fed different supplemental manganese levels of provided by organic and inorganic sources

Item	Manganese levels (mg/kg)					Source^1)^		SEM	p-value				
		
	0	25	50	75	100	Inorganic	Organic		Source	Level	Source ×level	L	Q
Breast muscle													
Superoxide dismutase (g/g pro)	2.10	2.08	2.13	2.14	2.06	2.15	2.05	0.027	0.081	0.864	0.731	0.879	0.487
Glutathione peroxidase (IU/g pro)	14,25	1,433	1,440	1,513	1,496	1,514	1,408	20.2	0.009	0.511	0.809	0.116	0.925
Liver													
Superoxide dismutase (g/g pro)	403	392	391	380	397	390	395	9.06	0.801	0.957	0.873	0.732	0.556
Glutathione peroxidase (IU/g pro)	30,516	30,534	30,344	30,557	30,665	30,897	30,149	159	0.018	0.978	0.158	0.770	0.656

SEM, standard error of means; L, linear effect of dietary manganese supplementation; Q, quadratic effect of dietary manganese supplementation.

1) Organic, manganese proteinate (MnPro) and organic trace minerals; inorganic: manganese sulphate (MnSO_4_·H_2_O) and inorganic trace minerals.

**Table 8:** Optimum supplemental manganese (Mn) levels for broiler chicks considering organic (MnPro) and inorganic (MnSO_4_·H_2_O) sources individually or combined

Item	Regression equations^1)^	Optimal Mn supplementation (mg/kg)	p-value	Coefficient of determination (R^2^)
Body weight^2)^ (g)				
Mn levels	BW = 549+19.3 (27.6–Mn)	27.6	<0.01	0.184
MnPro	BW = 549+24.2 (21.6–Mn)	21.6	<0.01	0.209
MnSO_4_·H_2_O	BW = 553+9.17 (59.9–Mn)	59.9	<0.01	0.188
Average daily gain^2)^ (g)				
Mn levels	ADG = 37.5+1.35 (27.6–Mn)	27.6	<0.01	0.186
MnPro	ADG = 37.5+1.81 (20.6–Mn)	20.6	<0.01	0.212
MnSO_4_·H_2_O	ADG = 37.8+0.632 (59.8–Mn)	59.8	<0.01	0.184
Feed conversion ratio^2)^ (g feed/g gain)				
Mn levels	FCR = 1.378–0.022 (61.3–Mn)	61.3	<0.01	0.213
MnPro	FCR = 1.373–0.036 (38–Mn)	38.0	<0.01	0.241
MnSO_4_·H_2_O	FCR = 1.368–0.018 (74.3–Mn)	74.3	<0.01	0.194
Mn balance^2)^ (%)				
Mn levels	Mn Balance = 35.87+1.9245 (18.58–Mn)	18.6	<0.01	0.597
ProMn	Mn Balance = 35.73+2.1455 (16.59–Mn)	16.6	<0.01	0.515
MnSO_4_·H_2_O	Mn Balance = 36.02+1.7448 (20.59–Mn)	20.6	<0.01	0.681
Breast muscle Mn^3)^ (concentration, mg/kg)				
Mn levels	Mn breast = −0.00003X^2^+0.00405X+0.5106	67.5	0.074	0.083
ProMn	Mn breast = −0.00005X^2^+0.00623X+0.4823	62.3	0.044	0.210
Liver Mn concentration^2)^ (mg/kg)				
Mn levels	Mn liver = 11.3+0.28725 (39.3–Mn)	39.3	<0.01	0.517
ProMn	Mn liver = 11.32+0.33763 (33.5–Mn)	33.5	<0.01	0.543
MnSO_4_·H_2_O	Mn liver = 11.25+0.26084 (43.1–Mn)	43.1	<0.01	0.517

BW, body weight; ADG, average daily gain; FCR, feed conversion ratio.

1) Regression equations obtained from fitting collected data.

2) Linear broken-line plateau model (LBL): Y = β0+β1×(β2–X), where (β2–X) = 0 for X>β2, Y is the dependent variable, X is the dietary manganese concentration (mg/kg), β0 is the Y value at the plateau, β1 is the slope and β2 is the Manganese concentration at the break point.

3) Quadratic polynomial regression model (QP): Y = β0+β1×X+β2×X2, where Y is the dependent variable, X is the dietary Manganese concentration, and β0 is the intercept, β1 and β2 are the linear and quadratic coefficients, respectively; maximum response concentration was obtained by: –β1(2×β2).
